# Preventive Effects of *Bacillus licheniformis* on Heat Stroke in Rats by Sustaining Intestinal Barrier Function and Modulating Gut Microbiota

**DOI:** 10.3389/fmicb.2021.630841

**Published:** 2021-04-06

**Authors:** Lei Li, Man Wang, Jikuai Chen, Zhuoran Xu, Shaokang Wang, Xinyu Xia, Dong Liu, Sheng Wang, Chaoyu Xie, Jianghong Wu, Jinfeng Li, Jiqianzhu Zhang, Meitang Wang, Jiangbo Zhu, Changquan Ling, Shuogui Xu

**Affiliations:** ^1^Department of Emergency, Changhai Hospital, Naval Medical University, Shanghai, China; ^2^School of Traditional Chinese Medicine, Naval Medical University, Shanghai, China; ^3^Department of Health Toxicology, Faculty of Naval Medicine, Naval Medical University, Shanghai, China; ^4^First Clinical Medical College, Southern Medical University, Guangzhou, China

**Keywords:** heat stroke, intestinal barrier, *Bacillus licheniformis*, probiotics, 16S rRNA, gut microbiota

## Abstract

Heat stroke (HS) models in rats are associated with severe intestinal injury, which is often considered as the key event at the onset of HS. Probiotics can regulate the gut microbiota by inhibiting the colonization of harmful bacteria and promoting the proliferation of beneficial bacteria. Here, we investigated the preventive effects of a probiotic *Bacillus licheniformis* strain (BL, CMCC 63516) on HS rats as well as its effects on intestinal barrier function and gut microbiota. All rats were randomly divided into four groups: control (Con) + PBS (pre-administration with 1 ml PBS twice a day for 7 days, without HS induction), Con + BL group (pre-administration with 1 ml 1 × 10^8^ CFU/ml BL twice a day for 7 days, without HS induction), HS + PBS (PBS, with HS induction), and HS + BL (BL, with HS induction). Before the study, the BL strain was identified by genomic DNA analysis. Experimental HS was induced by placing rats in a hot and humid chamber for 60 min until meeting the diagnostic criterion of HS onset. Body weight, core body temperature, survival rate, biochemical markers, inflammatory cytokines, and histopathology were investigated to evaluate the preventive effects of BL on HS. D-Lactate, I-FABP, endotoxin, and tight-junction proteins were investigated, and the fluorescein isothiocyanate-dextran (FD-4) test administered, to assess the degree of intestinal injury and integrity. Gut microbiota of rats in each group were analyzed by 16S rRNA sequencing. The results showed that pre-administration with BL significantly attenuated hyperthermia, reduced HS-induced death, alleviated multiple-organ injury, and decreased the levels of serum inflammatory cytokines. Furthermore, BL sustained the intestinal barrier integrity of HS rats by alleviating intestinal injury and improving tight junctions. We also found that BL significantly increased the ratios of two probiotic bacteria, *Lactobacillus* and *Lactococcus*. In addition, *Romboutsia*, a candidate biomarker for HS diagnosis, was unexpectedly detected. In summary, BL pre-administration for 7 days has preventative effects on HS that may be mediated by sustaining intestinal barrier function and modulating gut microbiota.

## Introduction

Heat stroke (HS) is a life-threatening disease characterized by extreme hyperthermia (usually core body temperature (Tc) > 40.5°C), central nervous system dysfunction, systemic inflammatory response syndrome (SIRS), and multiple-organ injury dysfunction syndrome (MODS) (Epstein and Yanovich, 2019). As estimated by climate models, heat waves are becoming more intense, more frequent, and longer lasting in the 21st century, resulting in the rapid increase of mortality risk of heat-related illnesses, of which HS is the most hazardous (Meehl and Tebaldi, 2004; Guo et al., 2017). Rapid cooling methods that help immediately lower the body temperature are the major emergency treatments to save patients. However, when people develop HS in areas where cooling is difficult to obtain, the (Tc) cannot be controlled at the early stage. Without effective first-aid measures, Tc can rise continually and lead to a cascade of events that includes SIRS, disseminated intravascular coagulation (DIC), MODS, and even death. At this stage, even if multiple adjuvant treatments and intensive care therapy are adopted, the case-fatality rate of HS patients is difficult to reduce and the occurrence of sequelae might be inevitable. Considering the lack of specific and effective drugs for HS, prevention is critical rather than treatment after onset (Epstein and Yanovich, 2019).

The gastrointestinal tract (GI), which needs functional intestinal barrier integrity and gut microbiota to perform properly, seems to be more sensitive to heat stress compared with other visceral organs, such as the liver, kidney, and lungs. Growing evidence indicates that intestinal injury caused by HS-induced visceral ischemia plays a key role in the pathogenesis and pathophysiology of HS (Lian et al., 2020; Ogden et al., 2020). Intestinal injury comprised of enterocyte death and tight-junction disintegration results in intestinal barrier dysfunction that triggers gut-derived endotoxemia, which activates subsequent systemic inflammatory responses and multiple-organ injury (Ye et al., 2019). Therefore, the intestines can be considered as the main route of HS progression, and sustaining the intestinal barrier might help prevent HS onset and subsequent pathology.

Probiotics are live microorganisms that, when administered in proper amounts, confer a health benefit on the host. Supplementation with probiotics can inhibit the colonization of pathogenic bacteria, improve gut barrier function, and improve gut flora (Hill et al., 2014). Recently, several studies have suggested that dietary probiotics can alleviate the adverse effects of stress. Notably, some *Bacillus* probiotic strains are able to fight against heat stress in poultry and in laboratory studies (Deng et al., 2012; Lei et al., 2013; Song et al., 2014). *Bacillus licheniformis* (BL) is a Gram-positive, spore-forming bacteria that has been widely applied in the livestock industry, and live BL powder (CMCC63516) is used as a treatment for gastrointestinal diseases and as a probiotic in China (Li et al., 2019). Since BL has probiotic benefits by enhancing intestinal barrier function and modulating gut flora, and because the intestine plays a key role in HS, we hypothesized that BL might ameliorate HS onset and alleviate HS progression by advanced short-term dietary application. To the best of our knowledge, there have been no studies on the preventive effects of BL against HS in rat models, and the influence of BL on gut microbiota has not been clarified. Thus, in the current study, we determined whether BL has preventive effects on HS rats and evaluated the influence of BL on the intestinal barrier and gut microbiota.

## Materials and Methods

### Animals

Adult male Sprague–Dawley (SD) rats, weighing 250–300 g, were purchased from Sippr B&K Laboratory Animal Ltd. (Shanghai, China). All rats were raised in the Specific Pathogen Free Animal Experiment Center of the Navy Medical University in China. The rats (6/cage) were maintained at an ambient temperature (Ta) of 22 ± 1°C and relative humidity (RH) of 50 ± 5% with a 12-h day/night cycle. Standard pellet rat chow and distilled tap water were provided *ad libitum*. All experimental procedures were approved by the Institutional Animal Ethics Committee of the Navy Medical University according to the Guide for the Care and Use of Laboratory Animals of the National Institutes of Health.

### Animal Grouping and Probiotic Administration

**FIGURE 1:**
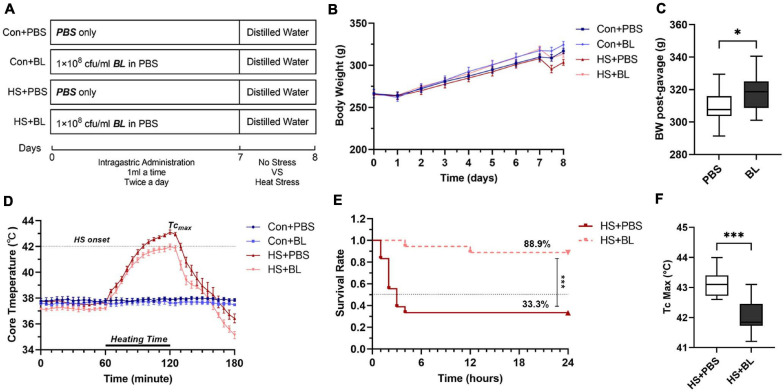
Experimental design and effects of *Bacillus licheniformis* (BL) on weight, core body temperature (Tc), and survival rate of rats with heat stroke (HS). **(A)** The BL group was administered 1 × 10^8^ CFU BL each twice a day for 7 days. After gavage, the HS model was induced by placing rats in an artificial climate chamber with Ta maintained at 40°C ± 1 and RH at 65% until the Tc of rats was beyond 42°C for approximately 60 min. **(B)** Body weight (BW) changes recorded every day are expressed from the starting gavage day. HS experiments were conducted just after the gavage stage, and pre-heating and post-heating BW were measured at day 7.5. **(C)** BW of post-gavage in the PBS groups was compared with BW in the BL groups. **(D)** Core body temperature (Tc, °C) was plotted for every 5 min from 1 h before heating to 1 h after heating. **(E)** Survival was monitored for 24 h after HS onset. **(F)** The maximum Tc in HS + PBS was compared with Tc Max in HS + BL. Data of Con + PBS (dark blue line with square), Con + BL (light blue line with circle), HS + PBS (dark red line with upright triangle), and HS + BL (light red line with inverted triangle) are plotted in **(B,D,E)**. Data in **(B,D)** are presented as the mean ± SEM of eight rats per group. Data in **(C,F)** are presented as a box-and-whisker plot of eight rats per group. Eighteen rats in each HS group were used for the survival study. ^∗^*P* < 0.05, ^∗∗∗^*P* < 0.001.

A total of 172 rats evaluated as suitable for research were randomly allocated into four groups by using a computerized randomization procedure. Rats in each group were processed according to uniform standards and then allocated to each experiment. The four groups were as follows: control + phosphate-buffered saline group (Con + PBS, *n* = 34); control + BL group (Con + BL, *n* = 34); HS + PBS group (HS + PBS, *n* = 52); and HS + BL group (Con + BL, *n* = 52). The whole research animal model process occurred in two stages that included intragastric administration and heat stress exposure (Figure 1A). In the first stage, rats were administered intragastrically with 1 ml PBS or 1 × 10^8^ colony-forming units (CFU) of BL (CMCC63516, Northeast Pharmaceutical Group, Shenyang No. 1 Pharmaceutical Co. Ltd., China, Lot S10950019) suspended in 1 ml PBS twice a day at fixed time points for 7 days (Li et al., 2019). Rats were weighed every day before gavage in case of stress-induced weight fluctuation. In the second stage, rats in the HS groups were exposed to heat stress, and rats in the Con groups were placed in a conventional environment without food and water.

### Heat Stroke Protocol and Core Body Temperature Measurements

Tc (±0.1°C) was monitored continuously at 5-min intervals in conscious, free-moving rats implanted with a temperature-monitoring capsule (SV223 capsule thermometer, Shenzhen Flamingo Technology Co., Ltd.). Briefly, each rat was anesthetized with isoflurane, and then an activated temperature-monitoring capsule, which was around 1.5 cm in length, 0.5 cm in diameter, and 2 g in weight, was surgically implanted intra-abdominally via a small incision with aseptic techniques. The capsule recorded Tc at 5-min intervals and sent the data to a cell phone by Bluetooth transmission. Due to the tiny size of the capsule and strong wound healing ability of rats, the physical habits of rats implanted with capsules were observed. To be included in the experiment, rats were implanted with capsules 1 week ahead of the study and their physical condition was confirmed as healthy with no abnormal behaviors (Helwig et al., 2012).

An artificial climate chamber (LTH-575N-01, Shanghai Drawell Scientific Instrument Co., Ltd.) where Ta was controlled at 40 ± 1°C and RH at 65 ± 5% to create an environment with high temperature and humidity was used to give rats heat stress (Xia et al., 2017). Rats in the HS groups were placed in the chamber at least 2 h a day during the first gavage stage to avoid stress-induced hyperthermia. To induce HS, rats were placed in a hot and humid chamber maintained at stable Ta and RH without food and water during the whole stage. Once rats were exposed to heat stress, Tc was monitored at 5-min intervals. The timepoint of stable hyperthermia occurrence (Tc > 42.7°C) was taken as the primary criterion of HS onset (Geng et al., 2015) and was allowed to continue for around 60 min with heating according to previous experience under our experimental environment. Then, rats meeting the criterion were transferred to regular housing with food and water access *ad libitum*. Body weights of rats in all groups were measured before and after the HS induction stage. Rats that died during or after this stage were recorded, and additional experiments were conducted to meet the sample size requirement. Overall, 36 rats in the HS + PBS and HS + BL groups each were used to observe and record survival within 24 h after the onset of HS.

### Sample Collection

Samples including serum, plasma, organ tissues, and feces were collected at 3 h after the onset of HS from rats anesthetized with isoflurane. Blood samples were collected from the abdominal aorta and separated by centrifugation at 3,000 rpm for 10 min at 4°C, then sub-packed and stored at −80°C for later examinations. Samples of liver, kidney, lung, and colon segments beside the ileocecal valve were harvested immediately after the blood collection and fixed in 4% paraformaldehyde for histopathological examination. Samples of colon specimens were cut into tiny parts that were fixed in 0.25% glutaraldehyde at 4°C for transmission electron microscopy. Fecal samples were quickly collected from colons from anesthetized rats and placed into liquid nitrogen to ensure their freshness for microbiological detection.

### Serum Biochemical Markers and Inflammatory Cytokine Analysis

Serum biochemical markers can represent the level of internal-organ injury. Alanine aminotransferase (ALT), aspartate aminotransferase (AST), blood urea (BU), creatine kinase (CK), and creatinine (CREA) were determined by a HITACHI 7080 automated analyzer (Tokyo, Japan). Serum inflammatory cytokines can represent the inflammatory level, so we detected the concentrations of IL-6, IL-1β, and TNF-α using a commercial Enzyme-Linked Immunosorbent Assay (ELISA) kit [Multi Sciences (Lianke) Biotech Co., Ltd., Hangzhou, China] and a SpectraMax M2e Microplate Spectrophotometer (Bio-Rad, Berkeley, CA, United States) according to the protocol provided (Xia et al., 2017).

### Histological Examination and Scoring

All the organ samples fixed in paraformaldehyde were dehydrated through a graded alcohol series and embedded in paraffin wax, sectioned into 10-mm sections, stained with hematoxylin and eosin (H&E), and examined under a light microscope (Leica DM 2000, Wetzlar, Germany). Selected H&E slides were scanned, and images were taken using a Panoramic MIDI Slide scanner (3DHISTECH, Hungary). Slide images were visualized by CaseViewer software (3DHISTECH). Then, histological profiles of organs in each group were analyzed with pathological injury scores. Typical pathological changes such as inflammation, necrosis, degeneration, hyperplasia, and fibrosis were described, and representative characteristics were chosen to construct an overall rating. Liver injury scoring was performed based on necrosis, inflammation, and vacuolar degeneration; kidney injury was based on congestion and inflammation; lung injury was based on alveolar wall thickening, congestion, and inflammation; and intestinal injury was based on necrosis, inflammation, and hemorrhage. The lesion degree of each kind of tissue was divided into five stages: no lesions or very few lesions, 0; mild or small lesions, 1; moderate lesions, 2; severe or multiple lesions, 3; and extremely severe or numerous lesions, 4; we then summarized each score for organs in each group rat for histopathological comparison.

### D-Lactate, I-FABP, and Endotoxin Detection

Plasma D-Lactate, intestinal fatty acid-binding protein (I-FABP), and endotoxin levels were, respectively, detected by a D-Lactate Colorimetric Assay Kit (BioVision, Milpitas, CA, United States), a Rat I-FABP ELISA Kit (F12794, YANJING Biological Co., Ltd., Shanghai, China), and a limulus amebocyte lysate (LAL) test kit (EC64405S, Xiamen Bioendo Technology Co., Ltd., Xiamen, China) according to the manufacturers’ instructions. The three detection kits all used 98-well plates with methods similar to the ELISA kit. Plasma samples and detection agents in each kit were mixed according to the provided instructions and measured at O.D. 450 and 545 nm in a microplate spectrophotometer. Standard curves were plotted, and then concentrations of each sample were calculated.

### FITC-Dextran 4-kD Detection

Intestinal barrier permeability was assessed using 4 kDa fluorescein isothiocyanate (FITC)-dextran (FD4, Sigma-Aldrich, St. Louis, MO, United States). In brief, a 200-mg/ml solution of FD4 was prepared with PBS and stored in a black Eppendorf tube. Then, the FD4 solution was orally administered to rats in each group at 200 mg/kg body weight, and rats were held in an upright position for 30 s to avoid regurgitation before the HS stage. Then, plasma samples were collected according to the study plan after the HS intervention and added to black 96-well microplates. Concentrations of FD4 were detected using a microplate spectrophotometer (excitation wavelength, 485 nm; emission wavelength, 530 nm). Concentrations of FD4 were calculated by preparing a standard curve with a serial dilution of FD4.

### Immunofluorescence Staining

Paraffin-embedded sections of the colon samples were also used for immunofluorescence staining. Samples were also obtained through deparaffinizing and rehydrating, antigen retrieval, circling, blocking endogenous peroxidase, blocking with serum, primary antibody (ZO-1, occludin, and E-cadherin) incubation, corresponding secondary antibody with HRP incubation, addition of TSA-FITC solution, microwave treatment, second primary antibody incubation, spontaneous fluorescence quenching, DAPI counterstaining of nuclei, mounting, microscopy detection, and image collection by fluorescent microscopy (NIKON ECLIPSE TI-SR, Tokyo, Japan). The image acquisition system (NIKON DS-U3) and microscope settings were maintained throughout the process.

### Transmission Electron Microscopy

Colon samples were quickly harvested and cut into small fresh tissues within 1–3 min for transmission electron microscopy (TEM). Colon tissue sizes were no more than 1 mm and were placed into fixative for TEM immediately. Colon tissues were kept in the dark and post-fixed with 1% OsO_4_ in 0.1 M PBS (pH 7.4) for 2 h at room temperature. After removal of OsO_4_, the tissues were rinsed in 0.1 M PBS (pH 7.4) three times for 15 min each. Then, samples were dehydrated with ethanol at room temperature, embedded in Epon-812 resin, and finally polymerized for more than 48 h at 65°C. Ultrathin sections (60–80 nm) were cut and stained with 2% uranyl acetate and 2.6% lead citrate (Xia et al., 2017). The sections were observed, and images were captured under a Hitachi-7000 electron microscope (Hitachi, Naka, Japan).

### Identification of *Bacillus licheniformis*

*Bacillus licheniformis*, which was stored as a spore powder, was provided by Northeast Pharmaceutical Group, Shenyang No. 1 Pharmaceutical Co. Ltd., China. The species-level identification of the *B. licheniformis* strain was conducted by genomic DNA analysis (Joseph et al., 2013; Selvarajan and Mohanasrinivasan, 2015). Total DNA of BL samples was extracted using a DNA purification kit (TIANamp Genomic DNA Kit, Tiangen Biotech CO., Ltd., China) according to the manufacturer’s instructions. PCR amplification of the 16s rDNA fragment was used for DNA amplification for 16S rRNA sequencing. The sequences obtained by 16S rRNA sequencing were subjected to a BLAST search^1^ of the NCBI database. Phylogenetic tree analysis was conducted on the NCBI website, and blast tree view was produced using BLAST pairwise alignments. Then, precisely designed amplified polymorphic DNA primers according to the genome sequence provided by the company (F 5′-GGTCGTATGCCTTCACCAGAT-3′ and R 5′-CGCTTTTTGCTCGGAAATGAT-3′) were used for polymerase chain reaction (PCR) amplification. An 807-bp PCR-amplified product was detected by agarose gel electrophoresis and analyzed to determine whether a sample was the BL strain (CMCC63516). Then, the amplified gene segment was subjected to a BLAST search in the NCBI database for blast tree view to confirm the species again.

### Fecal Microbiota Composition Analysis

Microbial community genomic DNA was extracted from 24 frozen fecal samples using the E.Z.N.A.^®^ soil DNA Kit (Omega Bio-tek, Norcross, GA, United States). The DNA concentration and purification were checked with a NanoDrop 2000 spectrophotometer (Thermo Scientific, Wilmington, DE, United States). The V3–V4 region of the bacterial 16S ribosomal RNA (rRNA) gene was amplified by PCR with primer pairs (338F 5′-ACTCCTACGGGAGGCAGCAG-3′ and 806R 5′-GGACTACHVGGGTWTCTAAT-3′) on an Applied Biosystems 7500 Real-Time PCR System (Life Technologies Corporation, United States) following the manufacturer’s protocols (Wang et al., 2020).

Amplicons were then purified by gel extraction with an AxyPrep DNA GelExtraction Kit (Axygen Biosciences, Union City, CA, United States) and quantified using a Quantus^TM^ Fluorometer (Promega, United States). Purified amplicons were pooled in equimolar amounts and subject to paired-end sequencing on an Illumina MiSeq platform (Illumina, San Diego, CA, United States) according to the standard protocols.

The raw 16S rRNA gene sequencing data were demultiplexed, quality-filtered by Trimmomatic, and merged by Fast Length Adjustment of Short reads (FLASH, v 1.2.11). Then, sequences were clustered into operational taxonomic units (OTUs) with a 97% similarity cutoff using UPARSE (version 7.1^2^), and chimeric sequences were identified and removed using UCHIME. The taxonomy of the acquired OTUs was analyzed by the RDP Classifier Bayesian algorithm^3^ against the SILVA (SSU138) 16S rRNA database with a confidence threshold of 0.7. Then, we subsampled each sample to an equal sequencing depth and clustered them for subsequent microbial bioinformatic analysis. The raw reads have been uploaded to the Sequence Read Archive (SRA) database (Accession: PRJNA674334 ID: 674334).

The 16Sr RNA sequencing data were analyzed using the Quantitative Insights Into Microbial Ecology platform (QIIME) with i-Sanger platform^4^ provided by Majorbio BioTech Co., Ltd. (Shanghai, China). Briefly, community diversity was evaluated using alpha diversity indexes that included species richness indices (Ace and Sobs) and species diversity indices (Shannon). Analysis of differences in beta-diversity as revealed by principal component analysis (PCA), principal coordinate analysis (PCoA), and non-metric multidimensional scaling analysis (NMDS) was conducted based on the OTU level from the weighted UniFrac distances. Differences between groups were tested by ANOSIM/Adonis, PERMANOVA, and partial least square discriminant analysis (PLS-DA). The Wilcoxon rank-sum test or Mann–Whitney *U* test was used to compare two targeted groups, and the Kruskal–Wallis *H* test was used to compare multiple groups at the phylum and genus levels. To investigate the effects of BL and HS on the gut bacterial communities in two groups, Student’s *t*-test was performed. A collinearity diagram was constructed by Circos software^5^ to visualize the corresponding abundance relationship between samples of each group and bacterial communities at the phylum and genus levels. Linear discriminant analysis (LDA) coupled with effect size measurements (LEfSe), which is an algorithm for biomarker discovery that identifies taxa characterizing the differences between two metadata classes, was used to determine differentially abundant features consistent with biologically meaningful categories among these groups. Species composition analyses were conducted based on the results of taxonomic analysis and are shown as bar graphs combined with statistical analysis.

### Statistical Analysis

All experimental data are presented as means ± standard error. All statistical analyses were performed with GraphPad Prism (Version 8.3.0, GraphPad Software, La Jolla, CA, United States), except the data of microbiota using multivariate and advanced statistical analysis, which is described above. Results from multiple groups were analyzed using one-way analysis of variance (ANOVA) followed by the Tukey–Kramer multiple-comparison test. Results from two unpaired groups were compared using the two-tailed Student’s *t*-test. Survival was analyzed with the log-rank test. Histological scores were analyzed by the Mann–Whitney rank-sum test. A two-tailed *P*-value of 0.05 or less was considered to be statistically significant.

## Results

### BL Pre-administration Attenuated Hyperthermia and Reduced HS-Induced Death

After the oral administration of BL, body weights (BW) of rats in the BL groups were heavier than in the PBS groups (Figure 1B). Combining the data of the PBS groups and BL groups, the BW post-gavage between the groups were found to be significantly different (Figure 1C; 309.0 ± 11.11 vs. 318.5 ± 10.77, *P* = 0.0201, 16 rats/group). Then, the HS rat model, which is widely used for research of HS, was used to evaluate the effects of BL on BW and Tc. The BW pre-gavage (Day 1), post-gavage (Day 7), and post-heating (Day 7.5) are shown in Table 1. Due to HS-induced fluid loss, the post-heating BW decreased around 10 g compared with the post-gavage BW but was not significant. Tc curves were plotted from 1 h before heating to 1 h after heating. All rats in the HS + PBS group (*n* = 8) met the HS onset standard (Tc > 42°C), while only two rats in the HS + BL group (*n* = 8) met the standard. The mean Tc of the HS + PBS group was higher than that of the HS + BL group as shown in Figure 1D, and the maximum Tc of rats in the BL groups was higher than in the PBS groups with significant difference (43.14 ± 0.46 vs. 42.01 ± 0.58, *P* = 0.008, *n* = 8 rats/group) as shown in Table 1 and Figure 1F. Furthermore, we found that the survival rate 24 h after HS onset was 33.3 and 88.9%, respectively, for the HS + PBS and HS + BL groups as shown in Table 1. The survival time and survival rate of the HS + BL group were notably improved by BL pre-administration compared with the HS + PBS group (log rank, *P* = 0.0003, 18 rats/group; Figure 1E).

**TABLE 1:** Experimental data and characteristics of rats subjected to BL and HS.

	Con		HS	
	PBS (*n* = 8 ∼ 10)	BL (*n* = 8 ∼ 10)	PBS (*n* = 8 ∼ 10)	BL (*n* = 8 ∼ 10)
BW pre-gavage, g	266.4 ± 14.43	266.4 ± 13.81	265.3 ± 11.63	265.4 ± 11.09
BW post-gavage, g	309.8 ± 11.01	317.5 ± 12.19	308.2 ± 11.91	319.4 ± 9.91
BW post-heating, g	–	–	285.6 ± 12.48	308.7 ± 11.33
BW lose-heating, g	–	–	12.6 ± 2.38	10.7 ± 2.65
Tc max, °C	–	–	43.1 ± 0.46	42.0 ± 0.58^#^
Time of HS onset, min	–	–	96.9 ± 6.51	113.3 ± 11.09^#^
ALT, U/L	25.2 ± 8.59	24.5 ± 6.47*	127.9 ± 36.17*	79.8 ± 15.6^#^
AST, U/L	81.8 ± 10.99	80.3 ± 11.44*	322.6 ± 60.27*	146.9 ± 34.07^#^
BU, mol/L	4.8 ± 2.26	5.3 ± 0.98*	19.5 ± 5.50*	14.2 ± 2.90^#^
CREA, μmol/L	6.7 ± 1.77	8.2 ± 1.44*	58.2 ± 18.17*	27.5 ± 7.054^#^
CK, U/L	291.3 ± 172.90	462.2 ± 116.10*	1631 ± 1002.00*	808.9 ± 336.60^#^
TNF-α, pg/ml	1.5 ± 0.31	1.1 ± 0.66*	3.3 ± 0.65*	1.8 ± 0.41^#^
IL-1β, pg/ml	5.9 ± 4.39	8.6 ± 4.54*	34.7 ± 6.69*	28.1 ± 3.94^#^
IL-6, pg/ml	4.0 ± 2.92	3.2 ± 1.92*	40.7 ± 10.87*	20.8 ± 4.41^#^
D-Lactate, μmol/mL	0.2 ± 0.03	0.2 ± 0.041*	0.7 ± 0.13*	0.3 ± 0.03^#^
I-FABP, pg/mL	216.3 ± 80.17	212.7 ± 63.04*	754.7 ± 266.2*	368.9 ± 117.3^#^
Endotoxin, EU/mL	0.1 ± 0.01	0.1 ± 0.01*	0.8 ± 0.13*	0.6 ± 0.13^#^
FITC-4kD, μmol/mL	0.1 ± 0.03	0.1 ± 0.04*	1.8 ± 0.86*	0.9 ± 0.43^#^

Data are shown as means ± SD; sample sizes are indicated in (n); BW, body weight; Tc max, mean value and SD of the maximum Tc during the heating period. Time of HS onset, the time from the beginning of heating to Tc reaching the standard of HS onset or to the end of the heating period in the BL group. ALT, alanine aminotransferase; AST, aspartate aminotransferase; BU, blood urea; CK, creatine kinase; CREA, creatinine; I-FABP, intestinal fatty acid-binding protein; FITC-4kD, 4-kDa fluorescein isothiocyanate. * vs. Con + PBS; ^#^ vs. HS + PBS.

### BL Pre-administration Attenuated Multiple-Organ Injury and Decreased Levels of Serum Inflammatory Cytokines

**FIGURE 2:**
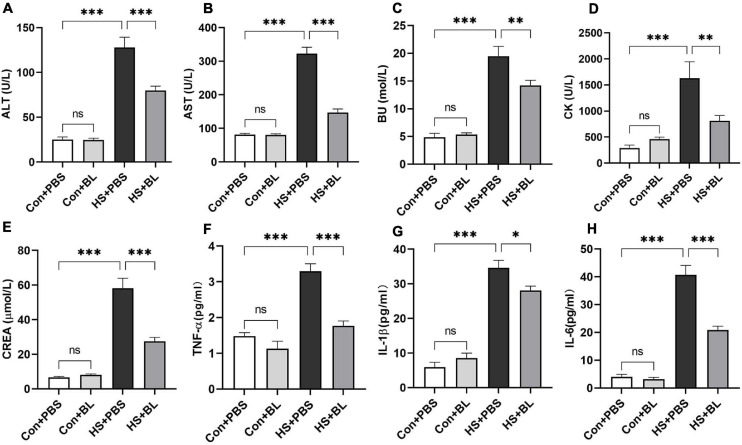
BL pre-administration decreased levels of serum biochemical markers and inflammatory cytokines. Rats were pre-administered with 1 × 10^8^ CFU twice a day (HS + BL group) for 7 days or PBS (HS + PBS group), and blood samples were collected 3 h after HS onset and at the same time for the control group rats. Serum biochemical marker levels of ALT **(A)**, AST **(B)**, BU **(C)**, CK **(D)**, and CREA **(E)** and inflammatory cytokines TNF-α **(F)**, IL-1β **(G)**, and IL-6 **(H)** are presented as means ± SEM, *n* = 10 per group. ^*ns*^*P* > 0.05, ^∗^*P* < 0.05, ^∗∗^*P* < 0.01, ^∗∗∗^*P* < 0.001. ALT, alanine aminotransferase; AST, aspartate aminotransferase; BU, blood urea; CK, creatine kinase; CREA, creatinine.

**FIGURE 3:**
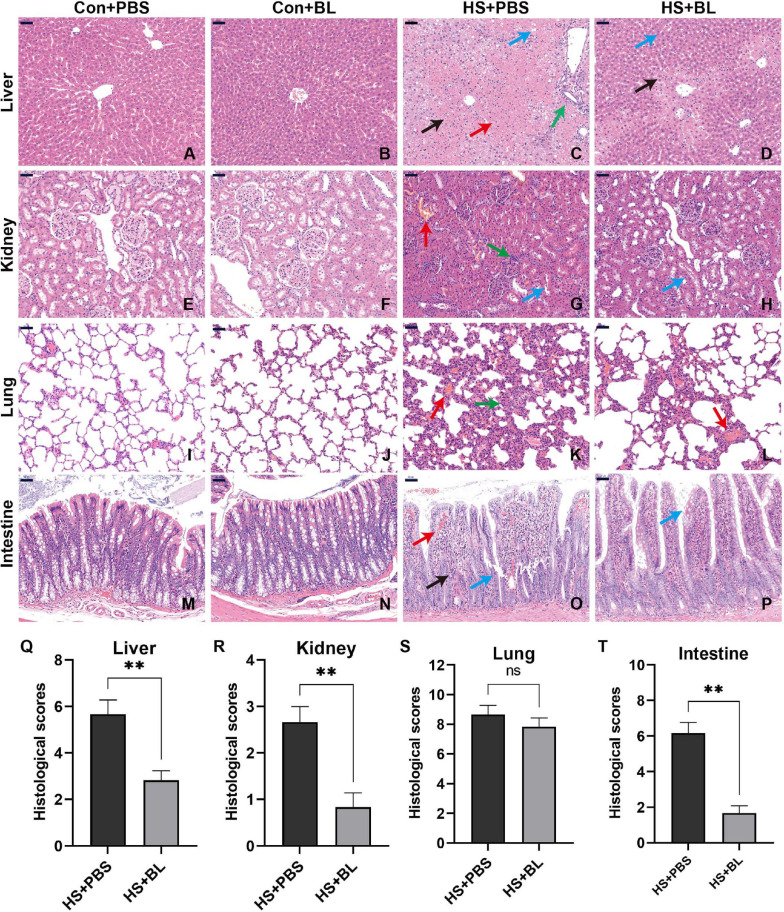
BL pre-administration attenuated multiple-organ injury in HS rats. Organ samples were harvested at 3 h after HS and stained with H&E. Representative histopathological images are shown of liver **(A–D)**, kidney **(E–H)**, lung **(I–L)**, and intestine **(M–P)** from Con + PBS, Con + BL, HS + PBS, and HS + BL (left to right) groups, magnification 200×. Scale bar = 50 μm. Arrows indicate typical pathological changes including necrosis, inflammation, vacuolar degeneration, congestion, hemorrhage, and alveolar wall thickening. Histological scores of liver **(Q)**, kidney **(R)**, lung **(S)**, and intestine **(T)** were counted and plotted. Values are presented as means ± SEM; *n* = 6 per group. ^*ns*^*P* > 0.05, ^∗∗^*P* < 0.01. Black arrows indicate necrosis; green arrows, inflammation; red arrows, congestion or hemorrhage; blue arrows, organ-specific alternations, such as villi destruction or hepatocyte vacuolar degeneration.

Multiple-organ injury in rats of each group was evaluated by checking biochemical markers that represent each related organ’s function and histopathological examination with H&E staining. Serum biochemical markers of liver function (ALT and AST), kidney function (BU and CREA), and skeletal and/or cardiac muscle function (CK) were chosen to evaluate typical organ injury. Typical serum inflammatory cytokines TNF-α, IL-1β, and IL-6 were selected to evaluate the inflammatory response. No statistically significant difference in these indexes was found between the Con + PBS and Con + BL groups, while levels of serum biochemical markers and inflammatory cytokines were notably increased in the HS groups compared with the Con groups with a significant difference as shown in Figures 2, 3. Consistent with the serum biochemical markers, livers from the HS + PBS group showed massive hepatocyte necrosis, hyperchromatic or cataclysmic nuclei, enhanced eosinophils, round vacuoles in the cytoplasm, and granulocyte infiltration around the local bile duct (Figure 3C, arrows). Typical kidney injuries were observed in the cortex of HS rats as characterized by edema, renal vesicle stenosis, granulocyte infiltration in the glomeruli, eosinophilic material in renal tubules, and congestion in the blood vessels (Figure 3G, arrows). Furthermore, massive alveolar wall thickening, narrowed alveolae, many monocytes scattered with granulocytes, and severe congestion in vessels were found in the lungs of HS rats (Figure 3K, arrows). Meanwhile, intestinal (colon) injuries were seen with extensive epithelial necrosis in mucosa marked with hyperchromatic or cataclysmic nuclei, subepithelial edema, congestion in most blood vessels, and destruction of villi structures (Figure 3O, arrows). However, BL pre-administration attenuated HS-induced multiple-organ injury, which was also reflected by biochemical markers (Figure 2) and histopathological images (Figure 3). All of these mitigative effects were statistically significant, except for the histopathological scores of lungs. Since BL pre-administration resulted in obvious preventive effects of organ injury, systematic inflammatory responses were checked by measuring the concentrations of inflammatory cytokines. Encouragingly, significant decreases of TNF-α, IL-1β, and IL-6 in the HS + BL group were found compared with the HS + PBS group. Altogether, this evidence indicated that BL pre-administration attenuates multiple-organ injury and significantly decreases the levels of serum inflammatory cytokines.

### BL Pre-administration Attenuated Intestinal Injury and Enhanced Intestinal Barrier Function

**FIGURE 4:**
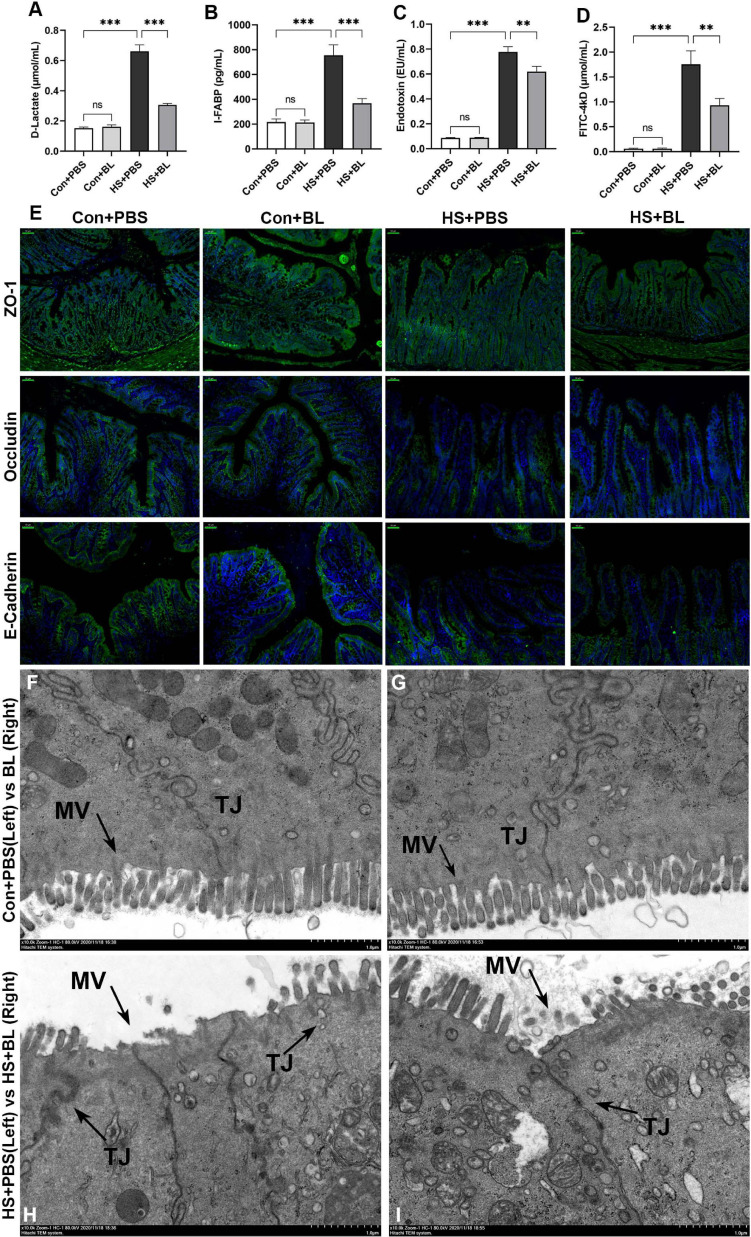
BL pre-administration attenuated intestinal injury and enhanced intestinal barrier function. Plasma D-Lactate **(A)**, I-FABP **(B)**, endotoxin **(C)**, and FD4 **(D)** were detected at 3 h after the HS onset in the Con + PBS, Con + BL, HS + PBS, and HS + BL groups. Concentrations are presented as means ± SEM; *n* = 10 per group. ^*ns*^*P* > 0.05, ^∗∗^*P* < 0.01, ^∗∗∗^*P* < 0.001. **(E)** Representative images of intestinal sections of rats from Con + PBS, Con + BL, HS + PBS, and HS + BL groups. Cell nuclei were stained using DAPI (blue), and ZO-1, occludin, and E-cadherin proteins were stained with corresponding antibodies (green). Scale bar = 50 μm, *n* = 6 per group. **(F–I)** Representative ultrastructural transmission electron photomicrographs of intestinal samples from Con + PBS **(F)**, Con + BL **(G)**, HS + PBS **(H)**, and HS + BL **(I)** show the morphology and sizes of cell nuclei, membrane microvilli (MV, arrows), and tight junctions (TJ, arrows). Scale bar = 1 μm. *n* = 3 per group.

As intestinal injury plays a key role in the progress of HS and might be the target organ protected by BL, typical biomarkers D-Lactate and I-FABP were chosen to evaluate the degree of intestinal injury. In addition, intestinal barrier function is highly associated with gastrointestinal permeability as determined by the level of plasma endotoxin and the FD4 test. Endotoxin escaping from the injured gut barrier might activate systematic inflammatory responses, resulting in multiple-organ injury. Significantly high levels of plasma D-lactate and I-FABP were detected in HS + PBS, while the levels of the two biomarkers were relatively low in HS + BL (Figures 4A,B). The initial degree of intestinal injury was thus lower in the HS + BL group. In addition, plasma endotoxin concentrations in the HS + BL group were lower than in the HS + PBS group with significant difference, which indicated that pre-administration of BL decreased intestinal barrier permeability (Figure 4C). Therefore, FD4 solution was pre-administered to rats for further investigation of intestinal permeability differences among groups. Once intestinal barrier dysfunction and permeability decrease occur, FD4 passes through the “leaky” gut into the blood and can be detected in the plasma. Quite high levels of FD4 were detected in the plasma of the HS + PBS group compared with Con + PBS, but relatively low FD4 levels were detected in the HS + BL group compared with HS + BL (Figure 4D). In addition, there was no significant difference of the above indexes between Con + PBS and Con + BL groups, which indicates that guts with or without BL both were healthy and “unleaky.” Representative proteins of tight junctions (TJs; ZO-1, occludin, and E-cadherin), which represent the permeability and function of the intestinal barrier, had a markedly lowered expression in the intestines of rats subjected to heat as shown in Figure 4E. Interestingly, the expressions of TJ markers were slightly increased in the rat in the Con + BL group, though the changes were subtle. However, the expressions of TJ markers were notably higher in the HS + BL group than in the HS + PBS group, which indicated that BL partly counteracted the HS-induced intestinal damage. To further explore the protective effects of BL on the gut, TEM was conducted to observe the ultrastructure of the intestinal alternations among groups. Consistent with the other results, we observed that BL pre-administration did not damage the intestinal barrier as represented by TJ structures and alleviated HS-induced intestinal injury. In brief, in the Con + PBS (Figure 4F) and Con + BL (Figure 4G) groups, intestine samples had similarly normal cell structures, mitochondria, and regularly aligned microvilli on the membranes, and clearly visible TJ structures between enterocytes, while in the HS + PBS (Figure 4H) group, intestine samples showed widespread damage to microvilli and TJ structure disruption as well as cell death. As expected, BL pre-administration attenuated these injuries and preserved intact TJ structures (Figure 4I). Taken together, the present data suggests that BL pre-administration alleviated intestinal injury and improved gut barrier function during and after HS onset.

### BL Pre-administration and HS Induction Modulated the Gut Microbiota Structure of Rats

**FIGURE 5:**
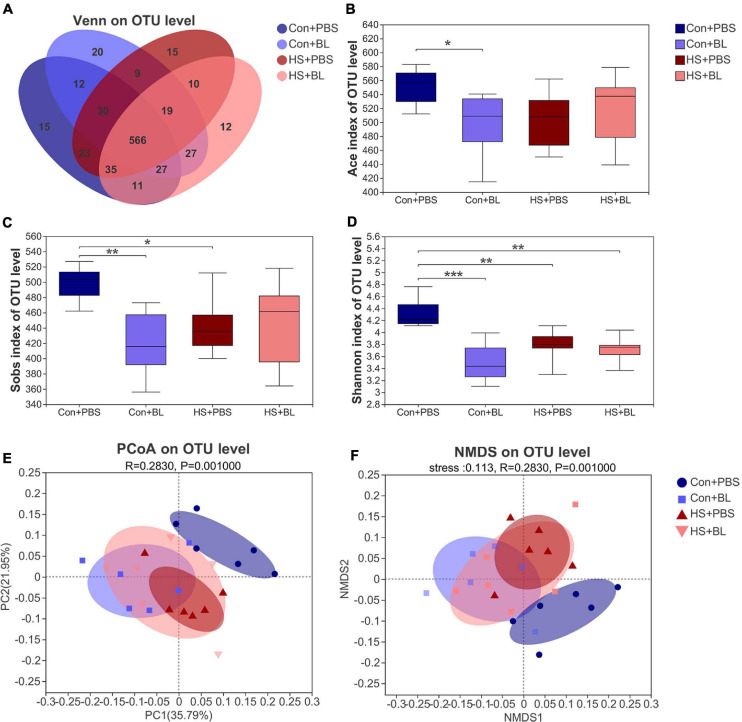
BL pre-administration and HS induction modulated the overall structure of gut microbiota of rats according to analysis of α-diversity and β-diversity. **(A)** Venn diagram of the operational taxonomic unit (OTU) distribution shows unique and shared OTUs between different experimental groups (*n* = 4). The Ace **(B)**, Sobs **(C)**, and Shannon **(D)** indexes of α-diversity analysis at the OTU level were calculated and compared among four groups. **(E,F)** β-Diversity was calculated by multivariate statistical analysis including principal coordinate analysis (PCoA), non-metric multidimensional scaling analysis (NMDS), and analysis of similarities (ANOSIM) at the OTU level based on weighted unifrac analysis, indicating significantly different gut microbiota structures among the four groups (ANOSIM, *R* = 0.283, *P* = 0.001). Data **(B–D)** are presented as a box-and-whisker plot of six rats per group. *P < 0.05, **P < 0.01, ***P < 0.001.

The gut microbiota from rat fecal samples in the four groups were investigated by analysis of the V3–V4 regions of 16S rRNA gene sequences to determine the composition of the gut bacterial community. A total of 835,056 sequences were obtained from 24 fecal samples from the four groups through size filtering, quality control, and chimera removal. Moreover, 831 operational taxonomic units (OTUs) including 280 species, 181 genera, 86 families, 52 orders, 20 classes, and 14 phyla of gut microbes from 24 samples were identified and annotated for subsequent analyses. The refraction curve of each sample from each group at the OTU level indicated that the sequencing data of these samples was sufficient to reflect the overall structure of the gut microbiota (Supplementary Figures 2C,D). A Venn diagram at the OTU level showed that, although the four groups shared 566 identical OTUs, each group still contained some unique bacteria (Figure 5A), and 15, 20, 15, and 12 unique OTUs were present in the Con + PBS, Con + BL, HS + PBS, and HS + BL groups, respectively. The richness of the microbial communities indicated by the ACE index (Figure 5B) and Sobs index (Figure 5C), and the diversity of microbial communities indicated by the Shannon index (Figure 5D), were reduced significantly at the OTU level by BL pre-administration and HS induction. Furthermore, significant differences (ANOSIM *R* = 0.283, *p* = 0.001) in β-diversity based on the weighted UniFrac distances among the four groups were determined and displayed by PCoA and NMDS at the OTU level (Figures 5E,F). The Con + PBS group was clearly separate from the Con + BL and HS + PBS groups, which indicated that BL pre-administration and HS induction altered the gut microbiota. Though the HS + BL group was not clearly separated from the Con + BL and HS + PBS groups, a certain degree of difference was observed among these groups. Moreover, samples from the Con + PBS, Con + BL, and HS + PBS groups were more clustered compared with the HS + BL group.

**FIGURE 6:**
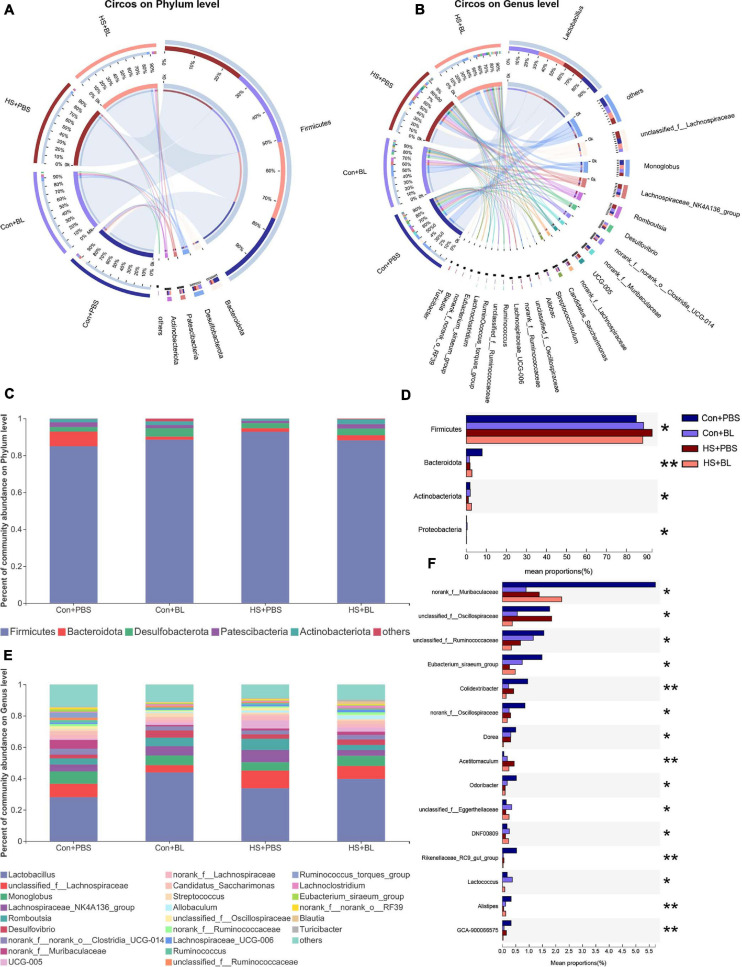
Alterations in the composition of gut microbiota in rats among each group. **(A,B)** Distribution of the microbial community for each group at the phylum and genus levels. The data were visualized by Circos software, and the width of the bars from each phylum or genus represents the relative abundance of that phylum or genus in this group. **(C,D)** Average relative abundances of microbial community composition for each group are shown by bar plots for the phylum level **(C)**, and a total of four phyla were found **(D)** with significant difference among the four groups. **(E,F)** Average relative abundances of microbial community composition for each group are shown by bar plots for the genus level **(E)**, and the top 15 abundant genera **(F)** with significant differences are shown among the four groups. Data are shown as the mean by bar plot analysis. *n* = 6 in each group. *P < 0.05, **P < 0.01.

**FIGURE 7:**
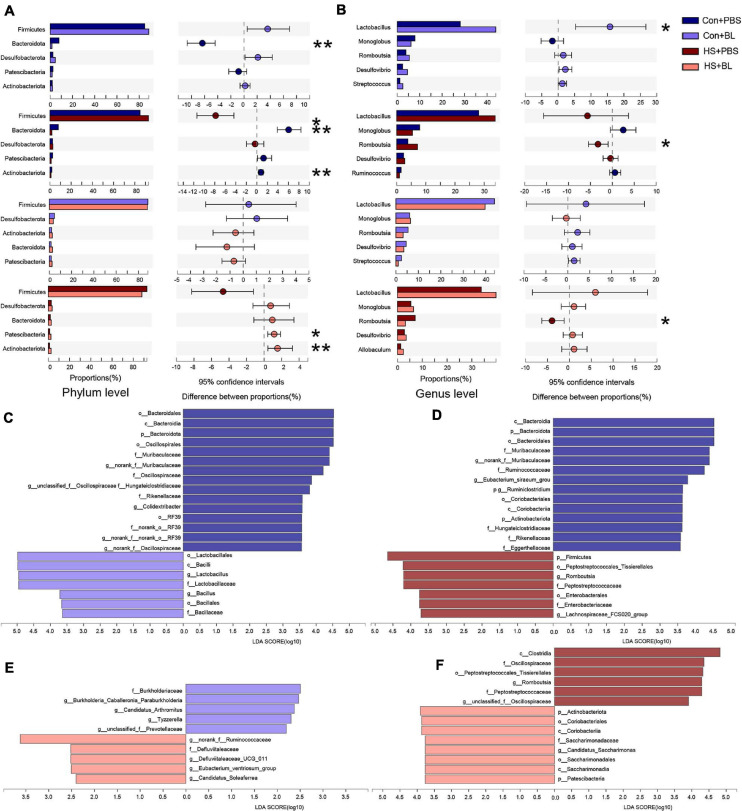
Pairwise comparisons of gut microbiota compositions between each group. **(A,B)** Mean relative abundances of the five most abundant phyla and genera (non-ranked or unclassified types were excluded) are shown by extended error bar plot and were compared between two groups at the phylum and genus levels. **(C–F)** Gut microbiota comparisons from phylum to genus between two groups by linear discriminant analysis effect size (LEfSe) analysis. Linear discriminant analysis (LDA) scores for the differentially abundant bacterial taxa between two groups were calculated by LEfSe to assess the effect size of each differentially abundant taxon. Only taxa having a *p*-value < 0.05 and LDA > 3.5 are shown in **(C,D,F)**, except **(E)** uses LDA > 2.0 due to the subtle difference in this comparison. Only taxa meeting an LDA significance threshold of 2 were included and shown. Enriched taxa in each group are indicated in red or blue. *n* = 6 in each group. *P < 0.05, **P < 0.01.

The gut microbiota community structure was illustrated by Circos and histograms (Figures 6A–C,E) at the phylum and genus levels, respectively. Then, multiple-group comparisons were conducted by the Kruskal–Wallis *H* test at the phylum and genus levels. A total of four phyla and the top 15 genera with significant differences were identified and are shown among the four groups (Figures 6D,F). Firmicutes, Bacteroidota, Desulfobacterota, Patescibacteria, and Actinobacteria were the predominant phyla in each group with relative abundances of >1%. *Lactobacillus*, *Monoglobus*, *Romboutsia*, and *Desulfovibrio* combined with 24 other genera were present in abundance in each group at the genus level. Though multiple-group comparisons identified that the average relative abundance in the microbial community composition for each group was notably different, partially distinguished microbiota compositions might be ignored or even not detected. Thus, eight pairwise comparisons of gut microbiota compositions were conducted to detect significant differences between groups subjected to BL or HS at the phylum and genus levels. The mean relative abundances of the five most abundant phyla and genera (non-ranked or unclassified types were excluded) are shown by an extended error bar plot (Figures 7A,B) and the significantly different phyla and genera are marked accordingly. In summary, following 7 days of BL pre-administration, BL-treated rats showed an increase in Firmicutes and a decrease in Bacteroidota at the phylum level. Correspondingly, the percent of *Lactobacillus*, which is a probiotic bacterium, was notably increased in BL-treated rats at the genus level. However, after HS induction, HS-treated rats had the same trend for Firmicutes and Bacteroidota on the phylum level with significant difference. Furthermore, the percent of *Romboutsia*, which is distinct and unreported in HS studies previously, was found to be increased in HS rats compared with Con rats. Interestingly, no significant difference was found between the Con + BL and HS + BL groups, which seemed to indicate that gut microbiota from BL-treated rats were not disturbed much by HS induction. To further determine the kinds of specific bacterial taxa in each group, the LEfSe analysis method, which uses LDA coupled with effect size analysis, was applied. Due to the number of differentially abundant phyla and genera, only taxa having *p*-values of <0.05 and LDA >3.5 are shown in Figures 7C,D,F, though Figure 7E shows LDA >2.0 due to the subtle difference in this comparison. The results of LEfSe analysis were basically consistent with the pairwise comparisons mentioned above and could identify multilevel species differences. In particular, the increase of *Bacillus* in BL rats, which is the genus of BL, confirms that BL colonized the guts of rats. Correspondingly, no differential species were determined for taxa of LDA >3.5 and only a tiny number of species were found when the LDA >2.0 between the Con + BL and HS + BL groups. These results collectively indicate that BL pre-administration and HS induction each modulated the gut microbiota of rats, and BL helped make the gut microbiota more probiotic and more heat-tolerable as characterized by the notable increase of *Lactobacillus* and the tiny alternations of gut microbiota of rats in the HS + BL group compared with the Con + BL group.

## Discussion

As the duration, intensity, and frequency of heat waves resulting from global warming increase, the incidence and mortality of HS are rapidly growing (Perkins-Kirkpatrick and Gibson, 2017). Prevention of HS is highly preferable to treatment. Therefore, alleviating intestinal injury and sustaining the intestinal barrier are conducive to the prevention of HS onset and its subsequent pathological cycle. BL pre-administration is a convenient and safe choice to prevent heat stress (Deng et al., 2012; Shing et al., 2014). However, the preventive effects of BL and its underlying mechanisms have yet to be determined. In this study, we demonstrated that BL pre-administration for 7 days exerted preventive effects on HS in rats, including attenuating hyperthermia, reducing HS-induced death, decreasing systematic inflammatory responses, and minimizing multiple-organ injury, by sustaining intestinal barrier function and modulating gut microbiota under HS.

Heat stroke is a life-threatening disease with severe outcomes if patients cannot be rapidly recognized and effectively treated. Although simple preventive and first-aid measures such as avoiding intense physical work in hot environments, conducting heat acclimation in advance, and medical rapid cooling are recommended (Liu et al., 2020), once HS occurs in a hot environment, rapid progression and limited medical treatment methods often lead to poor outcomes. The present understanding of the pathophysiology of HS indicates the need for mechanism-based intervention measures. In brief, heat exposure (from the environment) and/or exercise (from the body) can cause heat stress, which activates the thermoregulatory response. When excessive heat cannot be dissipated from the body and body temperature homeostasis cannot be maintained, the core body temperature rises continuously and stimulates multiple reflexive adjustments (Epstein and Roberts, 2011). Primarily, heat stress can produce direct thermal injury via heat cytotoxicity, acute-phase response, heat-shock response, and vascular endothelium damage. Secondly, to promote heat dissipation and reduce heat production, skin blood flow is increased to facilitate heat loss and visceral blood flow is decreased to compensate. Prolonged ischemia of visceral organs causes oxidative/nitrosative stress, and the gut, which needs functioning barrier integrity and microbiota to perform, is highly sensitive to heat stress and ischemia, so intestinal injury is found in the early stage of HS (Lian et al., 2020). As intestinal injury combined with a dysfunctional barrier and gastrointestinal microbial translocation from the “leaky” gut contributes to the pathophysiology of HS as characterized by SIRS and MODS, the etiology of intestinal barrier dysfunction following heat stress has become a focus of contemporary HS research (Hu et al., 2019). Therefore, countermeasures to maintain intestinal barrier integrity following heat stress are promising.

Probiotics are live microorganisms that confer a health benefit mainly through gut microbiota modulation, increased turnover of enterocytes, and competitive exclusion of pathogens (Liu et al., 2020). Each probiotic strain may generate species-level effects and strain-specific effects that include gut barrier reinforcement, nutrient production, and immunological effects. Recently, the administration of probiotics to sustain intestinal barrier integrity has been reported in several human studies, consistent with many *in vitro* and *in vivo* experiments (Hemert et al., 2013). Furthermore, several studies of poultry production have found that dietary supplementation of probiotics can alleviate heat stress-induced damage (Bron et al., 2012). A probiotics mixture containing *B. licheniformis*, *Bacillus subtilis*, and *Lactobacillus plantarum* has been found to increase growth performance, modulate intestinal microbiota, ameliorate jejunal morphology, and decrease the intestinal permeability of broilers subjected to heat stress (Song et al., 2014). Short-term administration of *B. subtilis* has been shown to prevent heat stress-induced adverse effects based on a simple rat model subjected to 45°C for 25 min (Sorokulova et al., 2016). Since some bacterial strains can modulate gut microbiota, sustain intestinal barrier integrity, and even fight against heat stress, combined with the advantages of safety, convenience, and accessibility, probiotics are a promising choice for the prevention and protection against HS. *B. licheniformis* (CMCC63516), a Gram-positive, spore-forming bacteria, was isolated from the vagina of a healthy woman in 1986 and has been used as an over-the-counter treatment for gut problems in China for more than 30 years (Li et al., 2019). It has been demonstrated that BL administration can counteract colon inflammation and gut dysbiosis caused by colitis in a mouse model, normalize the ileum microbiota of chickens with necrotic enteritis, and improve growth performance in broilers (Song et al., 2014; Xu et al., 2018). In brief, BL can sustain gut barrier function, modulate gut microbiota, counteract inflammatory responses caused by gut problems, and even improve growth. Moreover, BL, like other bacilli, can tolerate extreme environments and colonize in the gut easily and stably. In view of these advantages, we examined whether BL pre-administration could help prevent HS in rats. Since BL powder was obtained commercially, the probiotic benefits are probably from strain CMCC63516. Not all BL strains are non-pathogenic, and we performed genomic DNA analysis of the BL samples (Joseph et al., 2013; Selvarajan and Mohanasrinivasan, 2015). Sequencing of 16S rRNA and phylogenetics accompanied by BLAST searches of the NCBI database demonstrated that BL samples were of the phylum Firmicutes, class Bacilli, order Bacillales, family Bacillaceae, and genus *Bacillus*, showing more than 96% identity with several *B. licheniformis* strains (Supplementary Figure 1). An 807-bp PCR product, amplified with primers for the genome sequence provided by the company for identification, was detected by agarose gel electrophoresis. The amplified sequences separated by gel electrophoresis and sequenced were subjected to BLAST searches in the NCBI database using blast tree view to confirm the species, which provided similar results (Supplementary Figure 1D). After identification of BL, intragastric administration with 1 × 10^8^ CFU in 1 ml PBS twice a day at fixed timepoints for 7 days was used according to the literature and preliminary experimental results.

We established a classic HS rat model according to previous studies and made some improvements. We provided a high-temperature and high-humidity environment by building an artificial climate chamber, as we found that the long heating period in some studies might be attributed to the ambient environment without reaching the target parameters according to our experiments. In previous studies, many researchers have measured rectal temperature by inserting a thermometer into the rectum and monitoring Tc by timed measurements. However, the inserted length of the thermometer, operative skills, and stress-induced hyperthermia caused by temperature measurement can affect the Tc of rats significantly (Robertson et al., 2020). Therefore, in this study, an activated temperature monitoring capsule, which can record Tc in conscious and free-moving rats at 5-min intervals and transmit data, was surgically implanted intra-abdominally. We found that all adult male SD rats met the criterion (Tc > 42.7°C) during a 60-min heating period in the hot and humid chamber. So a 60-min heating protocol for HS induction was used in this study. When rats were exposed to heat stress, Tc rose rapidly at the beginning. When Tc was beyond 40°C, it escalated slowly but continuously. At the end of the heating period, the rats in the HS + PBS group all met the HS onset criterion, while only two rats in the HS + BL group met the standard. The maximum Tc in the HS + PBS group reached 43.1°C ± 0.46, which was significantly higher than in the HS + BL group (Table 1). Therefore, BL pre-administration attenuated HS-induced hyperthermia. Since uncontrolled hyperthermia in HS was the main cause of death, and this kind of hyperthermia was reduced by BL, the survival rates between HS + PBS and HS + BL groups were compared using a log-rank test. We found that the survival time and survival rate of the HS + BL group were notably improved by BL pre-administration. The present research indicates that systematic inflammatory response and multiple-organ injury are important driving factors of hyperthermia and death. We detected significant increases of biochemical markers of organ injury and serum inflammatory cytokines with visible histopathological injuries in the liver, kidney, lungs, and intestines of HS rats (Figures 2, 3), which is consistent with the pathophysiology of HS (Epstein and Roberts, 2011). This further validated our HS model. We found that BL pre-administration attenuated multiple-organ injury and decreased the levels of serum inflammatory cytokines significantly. Moreover, no evidence was found that BL pre-administration caused damage or inflammation. Taken together, our results preliminarily demonstrated that BL pre-administration prevents HS onset, alleviates HS-induced damage, and increases survival.

As mentioned above, the gut is considered the “motor” of SIRS, MODS, and other pathophysiological alternations in HS (Ogden et al., 2020). Visceral blood flow reduction caused by HS-induced blood redistribution can lead to intestinal ischemia and injury followed by intestinal barrier dysfunction and hyperpermeability. D-Lactate, which is normally maintained at a concentration of only about 0.01 mM, may be elevated in the plasma under various gastrointestinal conditions such as ischemia, short bowel syndrome, appendicitis, and Crohn’s disease, making it useful as a biomarker (Levitt and Levitt, 2020). I-FABP, which is solely expressed in the intestine and is released extracellularly after intestinal epithelial injury, can be a suitable early biomarker of intestinal injuries (Voth et al., 2017). Therefore, D-Lactate and I-FABP were chosen to evaluate HS-induced intestinal injury in the early stage. In this study, BL pre-administration showed no harm to the gut, while the two intestinal injury biomarkers were elevated in plasma with a significant difference in HS rats, consistent with H&E staining. As expected, BL pre-administration significantly decreased the levels of D-Lactate and I-FABP, which indicated that intestinal injury was attenuated. Intestinal injury can lead to intestinal barrier dysfunction, which generates a “leaky” gut permitting microorganisms and microbial products (e.g., endotoxin, flagellin, and bacterial DNA) to pass through. Of concern, lipopolysaccharides (LPS), also known as endotoxins, are the major component of the outer membranes of Gram-negative bacteria that are a large part of the gut microbiota. LPS can bind pathogen-associated molecular patterns to toll-like receptors (TLR), and TLR activation can initiate the production of many pro-inflammatory cytokines if too much LPS passes through the gut to the blood without effective detoxification (Liu et al., 2012; Guo et al., 2013; Rathinam et al., 2019). Therefore, the FD4 test and endotoxin detection were used to assess intestinal barrier permeability (Xia et al., 2017). Lower FD4 and endotoxin in the HS + BL group were detected than in the HS + PBS group, indicating that gut barrier integrity was sustained successfully by BL.

Tight junctions are dynamic structures with complex architecture that are composed of transmembrane barrier proteins (e.g., claudins, junctional adhesion molecules, occludin, and tricellulin) and cytoplasmic scaffolding proteins (e.g., the ZO family, cingulin, and afadin) (Dokladny et al., 2016). These proteins are directly connected with the intracellular cytoskeleton and linked to regulatory proteins. An injured intestine results in a “leaky” gut with increased intestinal permeability and decreased expression of TJ proteins as well as disrupted TJ structures. A dysfunctional intestinal barrier and disrupted TJ structures have been reported in many studies, which can be examined by immunofluorescence of TJ proteins and TEM of TJ structures (Uerlings et al., 2018; Hu et al., 2019; Koch et al., 2019). In the present study, similar alterations were detected in HS rats, represented as lower expression of TJ proteins ZO-1, occludin, and E-cadherin (Figure 4E), and as disrupted TJ structures, enterocytes, and microvilli in TEM images (Figures 4F–I). BL pre-administration significantly counteracted these effects and maintained the integrity of the intestinal barrier. Altogether, these results demonstrated that BL pre-administration can alleviate HS-induced intestinal injury and sustain intestinal integrity and function.

Previous studies have demonstrated that the spores of *Bacillus* can germinate, colonize, and act as a probiotic in the gut (Li et al., 2019). Our results indicated that BL pre-administration influenced the richness and diversity of gut microbiota compared with PBS as illustrated in Figure 5. However, we found that our BL pre-administration protocol downregulated richness and diversity in contrast to previous studies. We estimated that BL administered for 7 days might inhibit some harmful bacteria more effectively than promote desirable bacteria. According to PCoA and NMDS analysis (Figure 5), gut microbiota in the Con + PBS group were clearly distinguished from those in the Con + BL group, which demonstrated that BL pre-administration for 7 days modulated gut microbiota successfully. Surprisingly, gut microbiota in the Con + PBS group were distinguished from HS + PBS, which indicated that heat stress altered the gut microbiota. Despite many researchers having applied probiotics or prebiotics in poultry to help livestock fight heat stress, few have focused on the impact of heat stress on gut microbiota based on an HS model (Armstrong et al., 2018). In this study, the structures of gut microbiota in the HS + PBS and HS + BL groups could not be distinguished clearly, which indicates that the microbiota of BL rats can stay stable even with heat stress. The results from PCA and PLS-DA analyses imply a similar conclusion (Supplementary Figure 2).

Firmicutes and Bacteroidota account for more than 90% of the gut microbiota, and there was a significant increase in the ratio of Firmicutes in BL rats with a notable decreased ratio of Bacteroidota. BL belongs to the phylum Firmicutes, and this increase could be attributed to both the colonization of BL in the gut and the elevation of *Lactobacillus*. In fact, from the LEfSe analysis (Figure 7C), the proportion of the genus *Bacillus* was notably higher in BL rats than in PBS rats. This clearly demonstrated that BL colonized the guts of rats. BL, which is a Gram-positive and aerobic bacterium, can cause oxygen deprivation and promote the growth of anaerobic bacteria. *Lactobacillus*, which is a genus of Gram-positive, aerotolerant anaerobes or microaerophilic bacteria that belongs to the phylum Firmicutes, class Bacilli, order Lactobacillales, and family Lactobacillaceae, is a probiotic used for centuries (Zhang et al., 2018). Moreover, as shown in Figure 6F, *Lactococcus*, a genus of lactic acid bacteria, was also elevated significantly in BL rats and is one of the most important bacteria in the gut (Song et al., 2017). Studies have shown that *Lactobacillus* functions by maintaining the bacterial community in the gut, facilitating the absorption of nutrients and improving body immunity, and can even inhibit the inflammatory response in the gut by enhancing innate and adaptive immunity and defending against intestinal pathogens (Mu et al., 2018; Wang et al., 2018). *Lactococcus* species can produce antimicrobial peptides that have potent antibacterial effects against many Gram-negative bacteria, including *Staphylococcus*, *Listeria*, and *Clostridium* (Field et al., 2015). Altogether, the above results suggest that BL pre-administration might not increase the richness or diversity of gut microbiota but can significantly increase two kinds of important probiotics. Although several studies have investigated the impact of heat stress on gut microbiota, they have mainly focused on poultry production or agricultural animals such as hens, broilers, and pigs. There has been limited research based on rat models investigating HS and gut microbiota alterations.

Patients with HS who are treated in intensive care units may still die, and sepsis in the end-stage happens frequently in many case reports (Graber et al., 1971). Some doctors refer to HS as a sepsis-like disease. As bacteria and endotoxins originating from the gut are key exacerbating factors, we hypothesized that the high morbidity of HS might be related to specific pathogenic gut bacteria. We thus compared gut microbiota between Con + PBS and HS + PBS groups, and the results demonstrated that microbiota between the two groups were notably different (Figure 5). An analysis of their compositions showed that *Romboutsia ilealis* was significantly elevated in HS + PBS rats. We also compared the gut microbiota of Con + BL and HS + BL groups, but no significant difference was detected at the phylum or genus levels, and LEfSe analysis did not find any meaningfully different bacteria with LDA > 2.0. These results indicated that the gut microbiota of rats with BL pre-administration maintained stable compositions of gut bacteria after HS induction. Conversely, *Romboutsia* was also significantly elevated in the HS + BL group compared with the HS + BL group. Therefore, we speculated that *Romboutsia* might play a special role in the gut. *R. ilealis*, which is a Gram-positive obligately anaerobic bacterium and the dominant member of the ileal microbiota, is a strain that can utilize carbohydrates via different and partially redundant pathways and has not been proven harmful so far (Gerritsen et al., 2017, 2018). Limited research has demonstrated that *R. ilealis* is at a higher proportion in the gut microbiota of patients with neurodevelopmental disorders, type 2 diabetes, or dementia (Araos et al., 2018; Kang et al., 2019; Frazier and Leone, 2020). However, some researchers have classified *R. ilealis* as a probiotic listed with *Lactobacillus*, which implies that the elevation of *Lactobacillus* and *Romboutsia* may represent a kind of beneficial alteration of the gut (Wu et al., 2020). Interestingly, we observed a slight increase of the ratio of *Lactobacillus* in HS + PBS rats, though it was not significant. Some researchers have speculated that probiotics can promote the elevation of *Romboutsia*, but our results in BL rats do not support this hypothesis. Healthier rats in the HS + BL group did not show an increase in *Romboutsia*, in contrast to the rats in the HS + PBS group. Therefore, we speculate that the increased ratio of *Romboutsia* during the early stage of HS may represent an important signal indicating HS onset or rapid progression, and it may be used as a biomarker for early diagnosis. Any relationship between *Romboutsia* and subsequent pathology still needs further exploration and verification. Furthermore, in the present study, 16S rRNA sequencing allowed us to study alterations of the composition of the gut microbiota in each group and the potential beneficial effects. However, changes in the metabolites of gut flora should be investigated. Therefore, in future research, we will use two detection methods to uncover the mechanism of the preventive effects. First, untargeted metabolomics detection based on liquid chromatography–mass spectrometry will be conducted to compare metabolomics profiles of gut flora to discover how beneficial bacteria perform. Second, targeted metabolomics detection based on gas chromatography–mass spectrometry will be conducted to find alterations in the levels of short-chain fatty acids (SCFAs), as SCFAs are typical metabolites of gut bacteria that can sustain intestinal integrity and rehabilitate gut function (Chang et al., 2020). We believe that more explicit and distinct mechanistic explanations of this preventive effect will be clarified with the results of our planned experiments. If some potential significant metabolites can be detected, our team will verify their protective effects based on an intestinal epithelial cell barrier model of heat stress.

## Conclusion

In the present study, BL pre-administration for 7 days showed preventive effects against HS by attenuating hyperthermia, increasing survival rate, and alleviating systematic immune responses and multiple-organ injury. The preventive effects of BL may be mediated by sustaining intestinal barrier integrity and modulating gut microbiota. We demonstrated that BL modulates gut microbiota by upregulating probiotic bacteria strains and inhibiting harmful bacteria. Moreover, *Romboutsia*, a candidate biomarker for HS, was detected. Overall, our results demonstrated that 7 days of BL pre-administration prevented HS onset and alleviate its progression. Further studies of the use of probiotics for the prevention of HS are warranted.

## Data Availability Statement

The datasets generated for this study can be found in online repositories. The names of the repository/repositories and accession number(s) can be found in the article/Supplementary Material.

## Ethics Statement

The animal study was reviewed and approved by Institutional Animal Ethics Committee of the Navy Medical University.

## Author Contributions

SX, CL, and JBZ conceived the idea and designed the research. LL, MW, JC, ZX, SKW, XX, SW, CX, JW, JL, and JQZ performed the research and analyzed the data. LL, MW, and JC wrote the manuscript. DL, MTW, JBZ, CL, and SX have taken part in the revision of the manuscript. All authors read and approved the final version of the manuscript.

## Conflict of Interest

The authors declare that the research was conducted in the absence of any commercial or financial relationships that could be construed as a potential conflict of interest.

## Footnotes

1 https://pubmed.ncbi.nlm.nih.gov/

2 http://drive5.com/uparse/

3 http://rdp.cme.msu.edu/

4 http://www.i-sanger.com/

5 http://circos.ca/software/download/circos/

## Supplementary Material

Supplementary Figure 1 Species level identification of BL by genomic DNA analysis. **(A)** The obtained 16S rRNA sequences were subjected to BLAST searches in the NCBI database, and top 10 BLAST hits are shown. **(B)** BLAST hits on the query sequence for BL. **(C)** A BLAST tree view was produced using BLAST pairwise alignments for 16S rRNA of BL (using both neighbor-joining and maximum-likelihood algorithms). **(D)** Electrophoresis of BL samples after PCR amplification with special primers for identification, and BLAST pairwise alignments for the complete sequence of BL. Bar, 0.009 changes per nucleotide position.

Supplementary Figure 2 Species level identification of BL by 16S r RNA sequencing. **(A,B)** Pan and Core OTU analysis of samples from each group for evaluating whether the sample size for this sequencing is sufficient according to whether the Pan/Core species curve is flat. **(C,D)** The refraction curve of each sample from each group on OTU level indicated that the sequencing data of these samples was sufficient to reflect the overall structure of gut microbiota. **(E)** Analysis of differences in beta-diversity revealed by PCA. **(F–H)** Differences between groups were tested by ANOSIM/Adonis from weighted unifrac distances at the phylum and genus levels and by PLS-DA. Data in **(G,H)** are presented as a box-and-whiskers plot of six rats per group.

Supplementary Figure 3 Comparisons of gut microbiota compositions among the four groups. **(A)** Gut microbiota comparisons from phylum to genus among the four groups were analyzed by LEfSe. LDA scores for the bacterial taxa differentially abundant between two groups were calculated by LEfSe to assess the effect size of each differentially abundant taxon. Only taxa having a *p*-value < 0.05 and LDA > 3.5 are shown in the figure. **(B,C)** Average relative abundances of microbial community compositions for each group are shown by bar plots at the phylum and genus levels. The top 15 abundant phyla and genera are shown in the figure. Data are shown as the mean by bar plot analysis. *n* = 6 in each group. ^∗^*P* < 0.05, ^∗∗^*P* < 0.01, ^∗∗∗^*P* < 0.001.

Supplementary Figure 4 Summary of HS pathophysiological alterations that lead to a cascade of events including SIRS, DIC, MODS, and even death. Intestinal injury caused by HS-induced visceral ischemia plays a key role in HS pathogenesis and pathophysiology. Intestinal injury, which is comprised of enterocyte death and tight junction disintegration, results in intestinal barrier dysfunction and triggers gut-derived endotoxemia, which activates subsequent systemic inflammatory response and multiple organ injury.
