# Supplementary figures and images for: Preventive Effects of Bacillus licheniformis on Heat Stroke in Rats by Sustaining Intestinal Barrier Function and Modulating Gut Microbiota
Source: Front Microbiol. 2021 Apr 6;12:630841. doi: 10.3389/fmicb.2021.630841 (PMC8055866; doi:10.3389/fmicb.2021.630841)

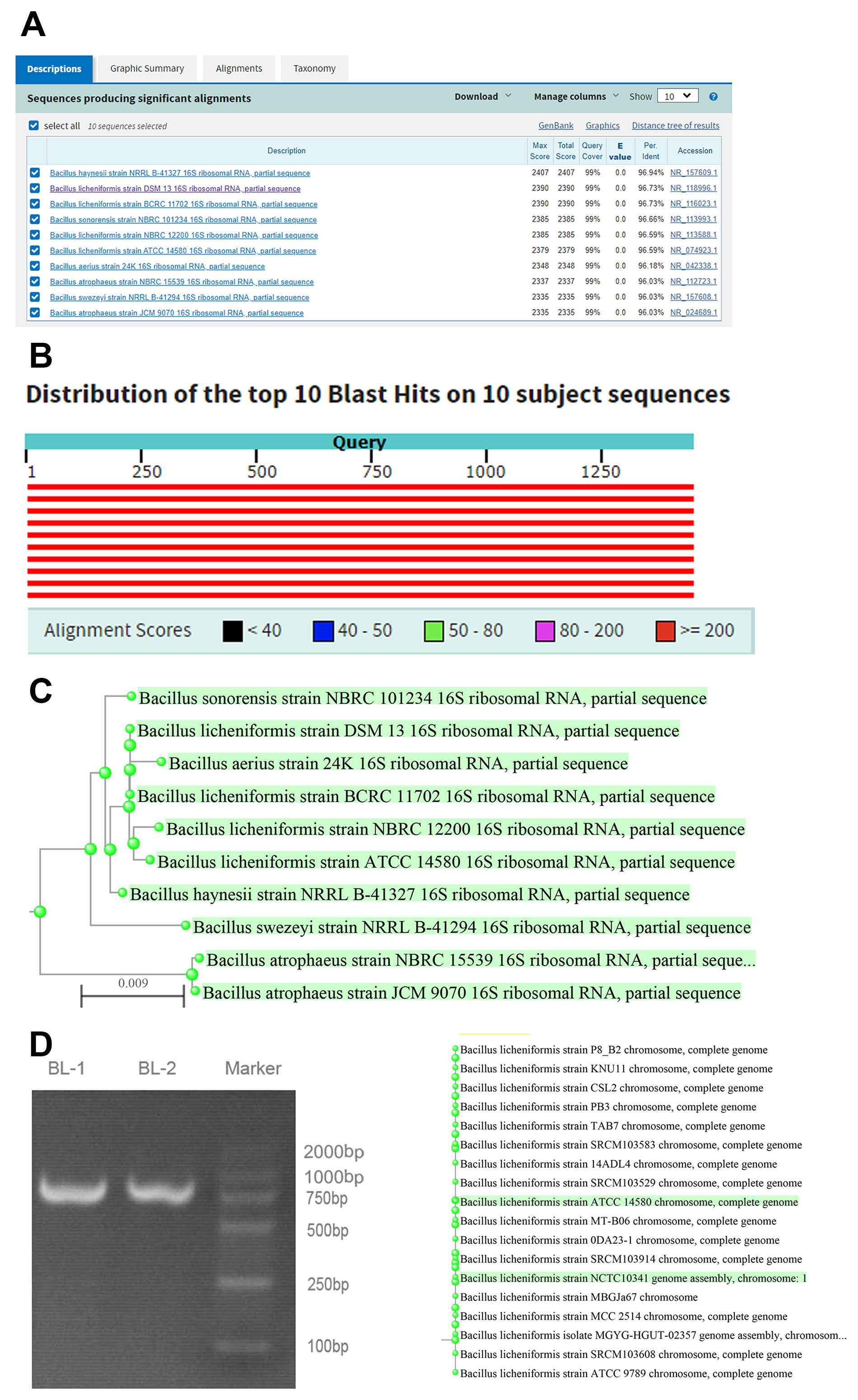

Supplement: Supplementary Figure 1 — Species level identification of BL by genomic DNA analysis. (A) The obtained 16S rRNA sequences were subjected to BLAST searches in the NCBI database, and top 10 BLAST hits are shown. (B) BLAST hits on the query sequence for BL. (C) A BLAST tree view was produced using BLAST pairwise alignments for 16S rRNA of BL (using both neighbor-joining and maximum-likelihood algorithms). (D) Electrophoresis of BL samples after PCR amplification with special primers for identification, and BLAST pairwise alignments for the complete sequence of BL. Bar, 0.009 changes per nucleotide position. [file Image_1.JPEG]

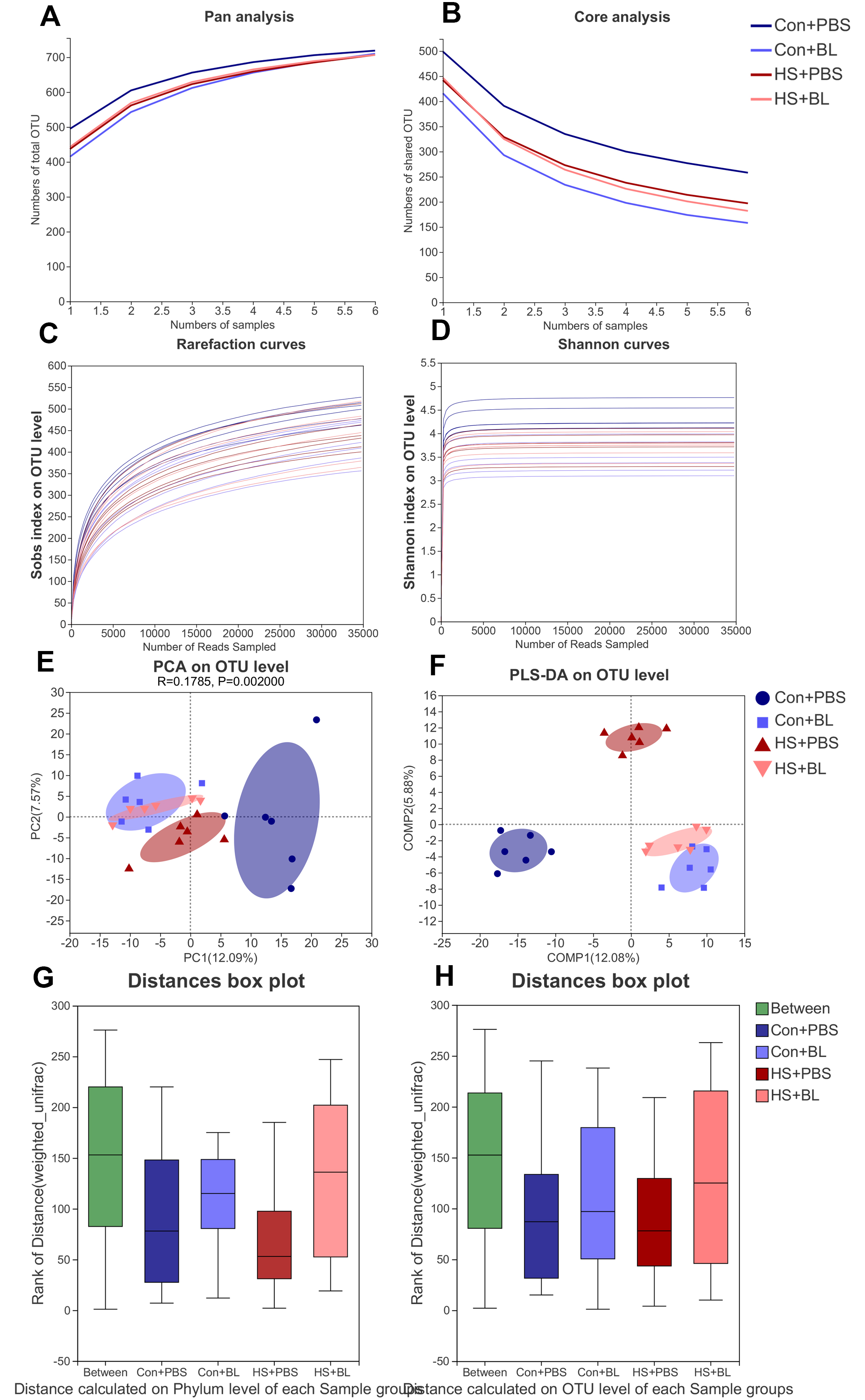

Supplement: Supplementary Figure 2 — Species level identification of BL by 16S r RNA sequencing. (A,B) Pan and Core OTU analysis of samples from each group for evaluating whether the sample size for this sequencing is sufficient according to whether the Pan/Core species curve is flat. (C,D) The refraction curve of each sample from each group on OTU level indicated that the sequencing data of these samples was sufficient to reflect the overall structure of gut microbiota. (E) Analysis of differences in beta-diversity revealed by PCA. (F–H) Differences between groups were tested by ANOSIM/Adonis from weighted unifrac distances at the phylum and genus levels and by PLS-DA. Data in (G,H) are presented as a box-and-whiskers plot of six rats per group. [file Image_2.JPEG]

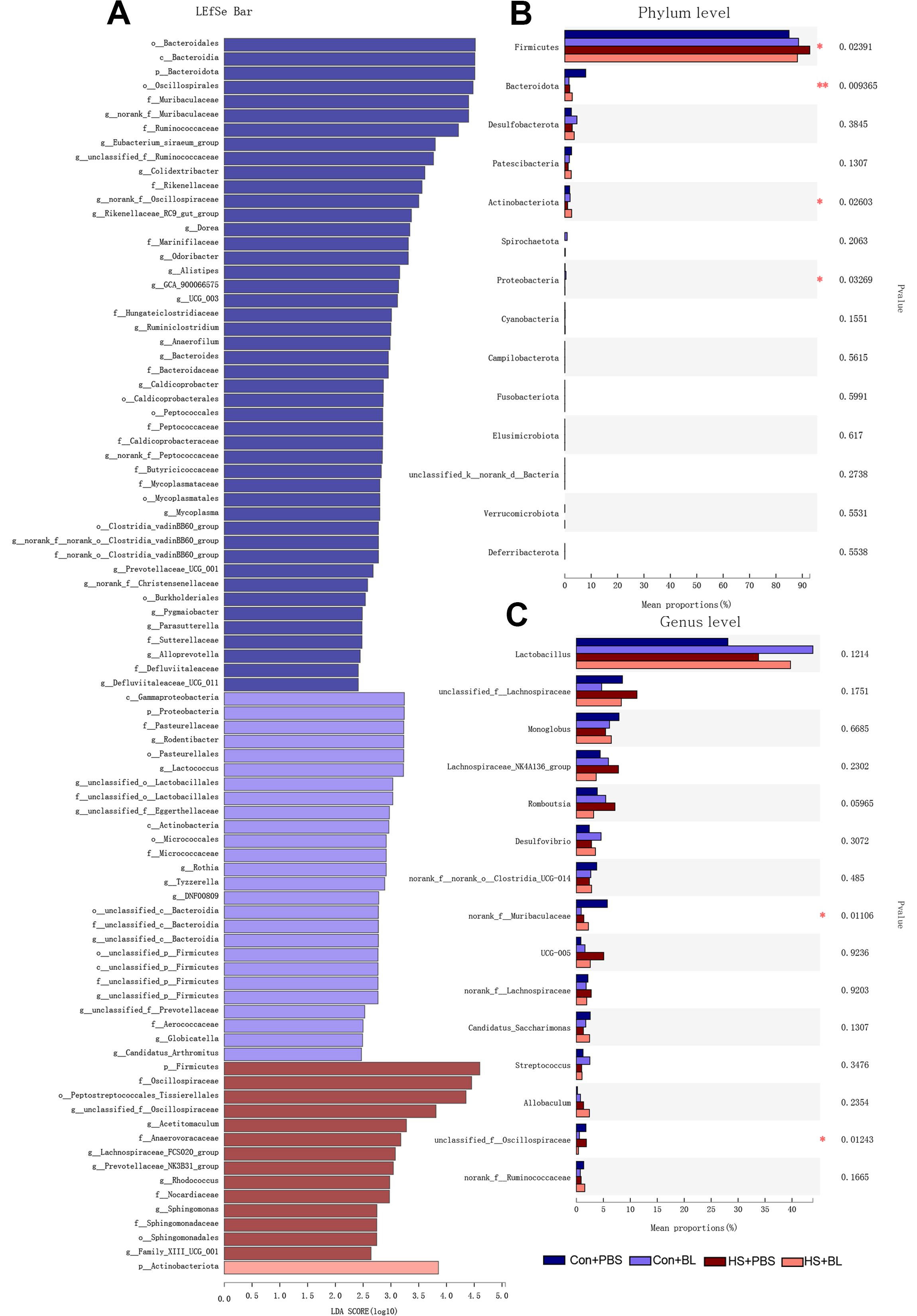

Supplement: Supplementary Figure 3 — Comparisons of gut microbiota compositions among the four groups. (A) Gut microbiota comparisons from phylum to genus among the four groups were analyzed by LEfSe. LDA scores for the bacterial taxa differentially abundant between two groups were calculated by LEfSe to assess the effect size of each differentially abundant taxon. Only taxa having a p-value < 0.05 and LDA > 3.5 are shown in the figure. (B,C) Average relative abundances of microbial community compositions for each group are shown by bar plots at the phylum and genus levels. The top 15 abundant phyla and genera are shown in the figure. Data are shown as the mean by bar plot analysis. n = 6 in each group. ∗P < 0.05, ∗∗P < 0.01, ∗∗∗P < 0.001. [file Image_3.JPEG]

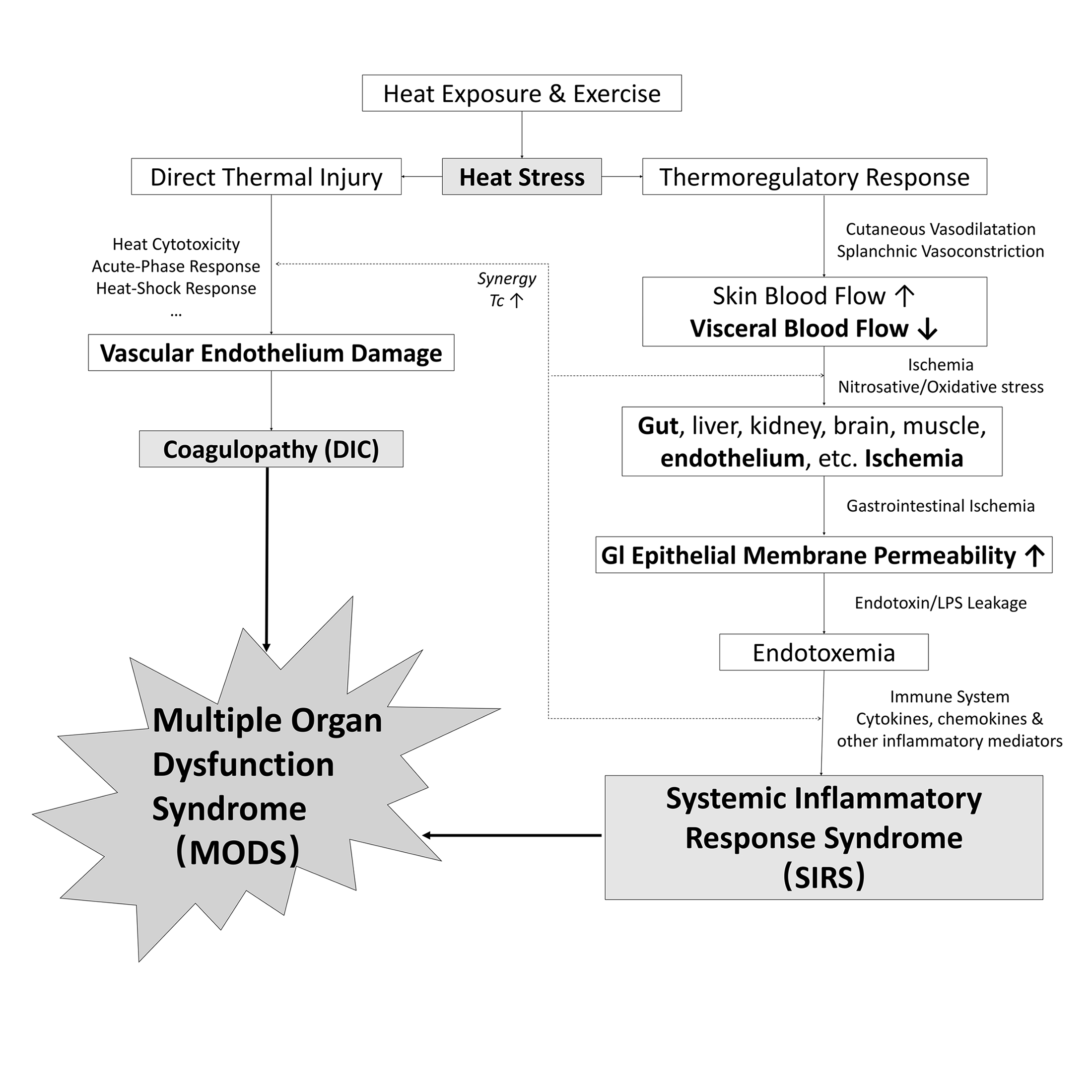

Supplement: Supplementary Figure 4 — Summary of HS pathophysiological alterations that lead to a cascade of events including SIRS, DIC, MODS, and even death. Intestinal injury caused by HS-induced visceral ischemia plays a key role in HS pathogenesis and pathophysiology. Intestinal injury, which is comprised of enterocyte death and tight junction disintegration, results in intestinal barrier dysfunction and triggers gut-derived endotoxemia, which activates subsequent systemic inflammatory response and multiple organ injury. [file Image_4.JPEG]
